# Nephron number variability in Japanese subjects: an autopsy-based study and its implications for chronic kidney disease: clinical scientist award address 2024

**DOI:** 10.1007/s10157-025-02662-3

**Published:** 2025-04-19

**Authors:** Go Kanzaki

**Affiliations:** Division of Nephrology and Hypertension, Department of Internal Medicine, The Jikei University School of Medicine, 3-25-8 Nishi-Shimbashi, Minato-Ku, Tokyo, 105-8461 Japan

**Keywords:** Nephron number, Glomerular density, Hyperfiltration, Hypertension, Chronic kidney disease

## Abstract

The number of nephrons is a key determinant of blood pressure regulation and chronic kidney disease (CKD) progression. Although traditional estimates suggest approximately one million nephrons per kidney, modern stereological approaches reveal substantial variability, that is influenced by ethnicity, birth weight, and other early life factors. This review evaluates the century-long evolution of nephron number research, variations across racial and ethnic groups, and explores how factors, such as body size, aging, and lifestyle risks, influence nephron endowment. Techniques for nephron quantification, from design-based stereology to emerging in vivo imaging, are also discussed. Recent research suggests markedly lower nephron counts in Japanese populations, especially among individuals with hypertension or CKD. The autopsy-based investigation in the present study included 27 middle-aged to older Japanese men (9 normotensive, 9 hypertensive, and 9 participants with CKD) who underwent dissector-fractionator stereology to quantify non-sclerosed glomeruli. Normotensive men had an average of approximately 640,399 non-sclerosed glomeruli. In contrast, the hypertensive participants had approximately 392,108 non-sclerosed glomeruli and those with CKD had only 268, 043. These findings underscore the potential influence of limited nephron reserves on hypertension and CKD in Japan. Current evidence suggests that nephron number estimates can guide therapeutic decisions and predict CKD outcomes, while advancements in real-time imaging offer potential avenues for non-invasive nephron assessment. Collectively, these developments highlight the central importance of nephron quantity in nephrology and enable targeted interventions aimed at preserving kidney function and mitigating the CKD burden.

## Introduction

Nephrons, the functional units of the kidney, are crucial for fluid balance, blood pressure regulation, and waste elimination. Their quantity significantly impacts hypertension and chronic kidney disease (CKD) risks, and they cannot regenerate once lost [1, 2]. While historical estimates suggest approximately 1 million nephrons per kidney, modern research shows individual variations ranging from under 200,000 to over 2 million [3, 4]. This variability raises questions regarding the factors determining nephron endowment and clinical implications.

Studies indicate that Japanese populations typically have fewer nephrons than Western populations, particularly those with hypertension or CKD [5]. Similar patterns exist in Australian Aboriginal populations, suggesting that genetic, developmental, and dietary factors may contribute to these differences [6].

Research has evolved from acid maceration to advanced stereological techniques, improving our understanding of nephron distribution across populations. This knowledge has become crucial for predicting disease progression, developing protective strategies, and improving transplant outcomes [7]. Factors affecting nephron numbers include birth weight, body size, and various environmental exposures, with the hyperfiltration theory of CKD highlighting their clinical relevance [8].

## Historical development of nephron number research

Early twentieth-century researchers pioneered nephron quantification through dissection and glomerular counting. The acid maceration technique, which involves dissolving kidney tissue to enable manual glomerular counting, provides the foundation for the widely cited estimate of one million nephrons per kidney [9]. Despite its relative simplicity, this method has several limitations including potential tissue distortion and incomplete glomerular separation. Nonetheless, acid maceration studies have established a widely disseminated figure of 1 million nephrons per kidney [10].

Research methods have subsequently evolved toward basic histological techniques, where tissue sections are stained and examined microscopically [11]. Although this two-dimensional approach reduced tissue destruction compared to acid maceration, it presented challenges in accurately counting glomeruli that spanned multiple sections. These limitations prompted the development of more precise counting methods.

Significant advances have been made with the introduction of design-based stereology [12]. By the 1970s and the 1980s, researchers began applying the disector and fractionator principles to obtain accurate and reproducible glomerular counts in postmortem kidneys. The disector principle enables comparison of parallel tissue sections to identify new glomeruli, whereas the fractionator principle provides a systematic sampling framework that divides the kidneys into hierarchical sampling levels. Together, these techniques allow for the calculation of total nephron number by multiplying the glomerular density within the cortical volume by the total cortical volume.

**Fig. 1:**
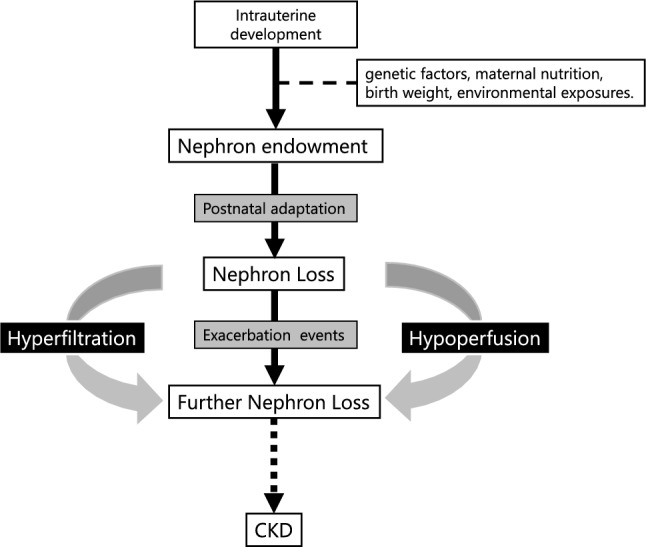
The progression of nephron reduction occurs through three key phases: intrauterine developmental programming, postnatal modification, and exacerbation. During intrauterine development, nephron endowment is influenced by multiple factors including genetic factors, maternal nutrition, birth weight, and environmental exposures. The postnatal phase involves continued modification of nephron structure and function, while the exacerbation phase establishes a self-reinforcing cycle where reduced nephron numbers lead to compensatory hyperfiltration by remaining glomeruli while simultaneously causing regional hypoperfusion. These aberrant hemodynamic patterns, characterized by excessive filtration pressure and inadequate blood flow, synergistically accelerate nephron loss and perpetuate a cycle of chronic kidney disease progression

Design-based stereology has become the gold standard for nephron counting in autopsied kidneys, significantly reducing bias and enabling correlation with clinical parameters [13]. Recently, model-based stereology has been proposed for in vivo nephron estimation by combining glomerular density from needle biopsy samples with cortical volume determined using computed tomography (CT) or magnetic resonance imaging (MRI) [14]. This method permits indirect estimation of nephron numbers in living individuals, although it requires invasive biopsies and meticulous volumetric analysis. Current research also explores advanced imaging techniques, including multiphoton microscopy and micro-CT, to non-invasively visualize glomeruli, although these methods remain in development and require further validation [15] (Fig. 1).

## Role of glomerular density in understanding number of nephrons

Glomerular density (GD) has emerged as a valuable independent measure of nephron masses in renal biopsy specimens. This parameter, calculated by counting the glomeruli within a defined cortical area, correlates well with the total number of nephrons and demonstrates strong associations with clinical outcomes across various glomerular diseases.

Tsuboi et al. showed that patients with immunoglobulin (Ig)A nephropathy who exhibit lower GD experience poorer long-term renal outcomes, suggesting that GD may serve as a predictor of disease progression and may help identify patients who require more intensive management [16]. Similar patterns have been observed in other glomerulopathies, including minimal-change nephrotic syndrome and membranous nephropathy [17, 18]. Koike et al. reported a relationship between GD, preterm birth, and gestational age in children with proteinuria, suggesting that perinatal factors can influence renal structure during childhood [19]. Kanzaki et al. focused on zonal differences within the renal cortex and noted that GD is not necessarily uniform across cortical regions, highlighting the importance of standardized sampling strategies [20].

Together, these studies emphasize that GD, an easily measured parameter in routine kidney biopsies, may help with direct nephron number estimates. Its predictive power in both adult and pediatric populations underscores its potential for guiding therapeutic decisions. Clinicians may use GD to assess the structural renal reserve and identify individuals who would benefit from stricter blood pressure control, protein restriction, or other renoprotective measures.

## Racial and ethnic variation in nephron number: global and Japanese insights

Australian Aboriginal communities have one of the world’s highest end-stage renal disease (ESRD) rates, with an average of approximately 680,000 nephrons per kidney, substantially below the traditional figure of one million common in Western populations [21, 22]. This shortfall, associated with factors such as poor prenatal nutrition and genetic vulnerability, also appears in certain African groups, albeit inconsistently [23, 24]. In the United States, studies on African Americans and White Americans underscore the complexity of directly linking nephron number to hypertension [25], and low nephron counts correlate strongly with hypertension in some White cohorts. However, African American populations do not always exhibit this pattern, possibly due to higher salt sensitivity, socioeconomic inequities, or other genetic modifiers [26].

The Japanese population has one of the highest dialysis-dependent CKD incidences, with recent autopsy investigations suggesting a pronounced deficit in nephron numbers [5]. In one study of 27 middle-aged to older males (consisting of 9 normotensive, 9 hypertensive, and 9 participants with CKD), normotensive participants averaged approximately 640,399 non-sclerosed glomeruli, whereas hypertensive participants had approximately 392,108, and those with CKD only 268,043. Single-nephron eGFR (SNeGFR) was also significantly higher in hypertensive (about 102.3 ± 23.9 nl/min) than in normotensive (about 71.9 ± 22.4 nl/min) participants, reflecting compensatory hyperfiltration. However, participants with CKD showed reduced SNeGFR relative to mean glomerular volume, suggesting that advanced vascular changes may limit hydrostatic pressure despite enlarged glomeruli. These findings indicate that a lower nephron baseline in Japanese individuals increases susceptibility to blood pressure elevations and CKD, particularly against a backdrop of nutritional deficiencies, aging, and vascular insults—factors that, when combined, may accelerate progression from hypertension to overt renal failure (Table 1).

**Table 1:** Nephron number in different racial groups

Population	Condition	*N*	Mean age	Male:female	Mean height (cm)	Approximate Mean nephron number	References
Japanese	Normotensive	9	64.1	10:0	163.2	640,000	[5]
	Hypertensive	9	68.3	10:0	164.7	390,000	
	Chronic kidney disease	9	71.1	10:0	164.4	270,000	
Australian Aboriginals	Normotensive + hypertensive	17	35.8	11:6	168.1	680,000	[6]
German	Normotensive	10	46.5	9:1	177	1,430,000	[21]
	Hypertensive	10	45.5	9:1	178	700,000	
African American	Normotensive	21	39.0	1.5:1	N/A	960,000	[23, 24]
	Hypertensive	41	47.4	1.3:1	N/A	860,000	
White American	Normotensive	36	45.8	1.3:1	N/A	920,000	
	Hypertensive	24	49.4	1.2:1	N/A	750,000	
Senegalese	Unknown	28	34.9	14:14	157.3	930,000	[25]

## Key factors influencing number of nephron

Multiple factors influence the nephron endowment and subsequent nephron loss [27]. Researchers have examined prenatal factors, postnatal growth, hypertension, obesity, diet, and aging in both humans and experimental models to better understand the etiology of low nephron number.

Birth weight is one of the most consistently documented determinants of nephron endowments [28, 29]. Epidemiological research has shown that infants with low birth weight exhibit lower nephron counts later in life [28]. The Barker hypothesis posits that inadequate fetal nutrition can lead to permanent structural changes and an increased risk of cardiovascular and renal diseases [30]. In regions such as Japan, where many older adults were born during World War II, maternal malnutrition and poor general health may limit renal development. Autopsy data from those born during or immediately after the war often reveal markedly reduced nephron numbers at an advanced age, aligning with an elevated risk of hypertension and chronic kidney disease [31]. In adulthood, obesity and a high-salt diet can intensify glomerular hyperfiltration and contribute to gradual attrition of functional nephrons. Excessive salt intake can aggravate hypertension, which imposes mechanical stress on the glomeruli, whereas obesity raises metabolic demands that push single-nephron filtration rates to damaging levels. Long-term exposure to these stressors hastens sclerosis in vulnerable glomeruli, further undermining the renal reserve [32].

Age-related nephron loss is another well-documented phenomenon [33]. Starting at middle age, the kidneys demonstrate measurable changes in structure and function, in part due to hypoperfusion, which contributes to a slow but steady decrease in the glomerular filtration rate. Histological studies have confirmed that kidneys of older individuals have fewer intact glomeruli, which aligns with the age-related reduction in total nephron number [14]. This depletion of nephron numbers with age occurs in conjunction with the onset of comorbidities, such as diabetes and atherosclerosis, compounding the risk of overt kidney disease.

Iatrogenic factors also contribute to decreased nephron count [34]. Repeated use of nephrotoxic medications, including non-steroidal anti-inflammatory drugs and certain antibiotics, can induce acute kidney injury and accelerate chronic injury over time. Contrast media used in radiographic procedures poses a further risk, especially in individuals who already have reduced nephron numbers. Over many years, these recurring insults have confounded pre-existing vulnerabilities and set the stage for progressive decline in renal function.

## Application of number of nephrons insights to clinical practice

Understanding the number of nephrons in an individual patient or population can help to elucidate the mechanisms of hypertension, guide risk stratification for CKD, and inform therapeutic interventions. A low nephron count indicates that the remaining nephrons must handle a disproportionate share of the total filtration burden. This phenomenon, referred to as hyperfiltration, increases intraglomerular pressure, leading to structural changes and eventual nephron loss through sclerosis [35].

The concept of hyperfiltration has evolved to distinguish between renal hyperfiltration, which involves the entire organ, and glomerular hyperfiltration, which focuses on single-nephron function. The single-nephron glomerular filtration rate (SNGFR) was calculated by dividing the total glomerular filtration rate by the estimated number of nephrons [36]. In settings where the number of nephrons is substantially reduced, each nephron works harder, increasing SNGFR and leading to microvascular damage, podocyte stress, and eventual fibrotic changes in the glomerulus [37]. Studies in Japanese cohorts have shown that individuals with hypertension often have elevated SNGFR levels, which is a sign of compensatory hyperfiltration [5]. Individuals with advanced CKD can exhibit variable SNGFR values, because some nephrons remain hyperfiltered, whereas others approach filtration failure. These findings highlight the dynamic and multifaceted nature of nephron function during disease progression.

Efforts to estimate the number of nephrons in the clinical setting have expanded rapidly. One approach involves collecting small tissue samples by kidney biopsy, staining them to identify glomeruli, and then applying stereological methods to measure GD. Researchers have simultaneously measured the cortical volume of the same kidney using unenhanced and contrast-enhanced CT imaging [14, 38, 39]. The product of GD and cortical volume provides a fairly accurate nephron count estimate that can help clinicians determine how aggressively to manage a patient’s risk factors, including hypertension management and protein intake [40]. Although not yet universally available, emerging imaging-based techniques promise more direct means of quantifying nephrons in vivo.

Haruhara et al. also explored podocyte number and its correlation with nephron endowment [41]. Podocytes are terminally differentiated cells that play a central role in maintaining glomerular filtration barrier integrity. Podocyte depletion is an early hallmark of progressive kidney diseases. A close relationship between nephron count and podocyte number has been reported, suggesting that strategies that preserve podocyte health may help mitigate the negative consequences of limited nephron reserves [42].

Clinical trials and observational studies further suggest that diet modulation, particularly protein intake limitation, can alter SNGFR and potentially slow kidney damage in individuals with low nephron counts. The protective benefits of lowering intraglomerular pressure using agents that block the renin–angiotensin–aldosterone system were also observed in patients with higher SNGFRs. These pharmacological interventions may disrupt the hyperfiltration cycle and cause nephron loss.

## Future directions for measuring and leveraging the number of nephron

Although autopsy studies remain the most accurate means of counting nephrons through design-based stereology, several evolving methods have aimed to achieve a robust estimation of the number of nephrons in living individuals. Model-based stereology has already been used in specialized centers, combining glomerular density from biopsy samples with cortical volume from imaging to provide an overall nephron estimate. As imaging technology improves, standard CT or MRI scans may provide higher-resolution data that reduce the reliance on invasive biopsies [43].

Efforts to refine these methodologies have led to investigations using novel imaging techniques, such as multiphoton microscopy, optical coherence tomography, and advanced contrast agents. Early research also explored micro-CT scanning in rodent kidneys, which achieved exceptionally detailed visualization of the glomeruli [44]. Translating such methods into a human clinical setting requires addressing the challenges related to resolution, radiation exposure, and contrast agent safety.

Public health and health policy initiatives in Japan and other countries with lower birth weights should emphasize improving maternal nutrition and reducing prenatal risk factors to promote healthy fetal development [45]. Decreasing the prevalence of low birth weight would likely help to ensure a more robust nephron endowment in the population. Dietary and lifestyle interventions, such as lowering excessive salt intake, managing body weight, and avoiding repeated nephrotoxic insults, have the potential to prevent premature nephron loss in adulthood.

A better understanding of nephron variability and its interplay with hypertension and CKD also provides potential opportunities for personalized medicine. As investigators continue to refine equations that incorporate renal size, glomerular density, and demographic factors, clinicians may soon be able to estimate nephron numbers with sufficient accuracy to tailor their management strategies. Ideally, such data would help identify individuals who require more aggressive blood pressure control or specific dietary modifications to protect their renal reserves.

## Conclusion

Recent analyses show significant nephron number variation across populations, with Japanese cohorts having lower counts. This correlates with higher hypertension and chronic kidney disease risks through hyperfiltration mechanisms. Clinical nephron quantification enables better risk assessment and treatment optimization. Future priorities include improving measurement methods, understanding disease mechanisms, and developing evidence-based protocols to preserve kidney function and enhance patient outcomes.
